# Cancer du sein inflammatoire

**DOI:** 10.11604/pamj.2016.23.260.9055

**Published:** 2016-04-29

**Authors:** Sami Aziz Brahm, Fatima Zahra Ziani

**Affiliations:** 1 Service d'Oncologie Médicale, Centre Hospitalier Mohammed VI, Oujda, Maroc; 2 Service d'Oncologie Médicale, Centre Hospitalier, Hassan II, Fès, Maroc

**Keywords:** Cancer du sein, inflamation, diagnostic précoce, Breast cancer, inflammatory, early diagnosis

## Image en médecine

Le cancer du sein inflammatoire est une entité clinique s'individualisant au sein des cancers mammaires par une épidémiologie, des critères diagnostiques et un pronostic spécifiques. Il est relativement rare et représente entre 1 et 5% des cancers mammaires. Le diagnostic du cancer du sein inflammatoire repose essentiellement sur des critères cliniques; La définition la plus communément admise, est celle de l'American Joint Committee of Cancer (AJCC) qui le décrit comme étant une entité clinicopathologique caractérisée par un érythème diffus associé à un œdème, souvent sans masse palpable sous-jacente. Dans sa forme complète, le sein tumoral est gros, lourd, tendu, douloureux, chaud, érythémateux, et la palpation peut ne pas individualiser de tumeur. Nous présentons le cas d'une patiente âgé de 45 ans qui a présenté deux mois avant la consultation, l'apparition d'une rougeur du sein avec douleur sans nodule palpable. La biopsie a révélé un carcinome canalaire infiltrant avec présence d'emboles vasculaire au niveau dermique. Le praticien doit être en mesure de reconnaitre ce diagnostic pour demander les examens complémentaires adéquats et référer la patiente pour une prise en charge rapide vu le risque métastatique accru de cette maladie.

**Figure 1:**
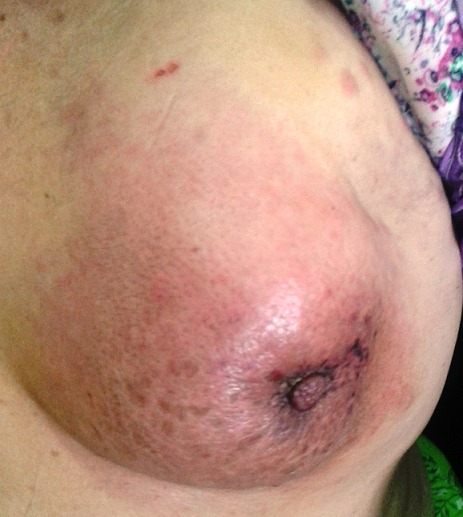
Image montrant un sein gauche avec un érythème diffu

